# Trends in the incidence of acute watery diarrhoea in the Lao People's Democratic Republic, 2009–2013

**DOI:** 10.5365/WPSAR.2016.7.2.006

**Published:** 2016-09-30

**Authors:** Souphatsone Houatthongkham, Noikaseumsy Sithivong, Gregory Jennings, Manilay Phengxay, Phanthaneeya Teepruksa, Bouaphanh Khamphaphongphane, Phengta Vongphrachanh, Kongmany Southalack, Dapeng Luo, Cindy H Chiu

**Affiliations:** a National Center for Laboratory and Epidemiology.; b World Health Organization, Lao People’s Democratic Republic.

## Abstract

Diarrhoeal disease is the second leading cause of death in children under age 5 worldwide, with rotavirus being the main etiology. In the Lao People's Democratic Republic, acute watery diarrhoea (AWD) was introduced as one of the national notifiable diseases in 2004. We retrospectively reviewed the aggregate (*n* = 117 277) and case-based (*n* = 67 755) AWD surveillance data from 2009 to 2013 reported weekly from 1115 health facilities nationwide. Rotavirus rapid test data from all eight sentinel sites in Vientiane Capital in 2013 were also collected for analysis. The incidence of AWD ranged between 215 and 476 cases per 100 000 population and increased from 2009 to 2012 when it levelled off. The most affected age group was children under 5 who were about seven to nine times more likely to have AWD than the rest of the population (*P* < 0.0001). In children under 5, 74.8% of the cases were aged 0–24 months and AWD was 1.28 times more common in males (*P* < 0.0001). Among the 230 stool specimens tested in children under 5 in 2013, 109 (47.4%) tested positive for rotavirus. The increased AWD incidence over the study period may reflect a true increase in AWD or an improved sensitivity of the system. We recommend new mothers breastfeed up to two years after birth, which is known to reduce AWD morbidity and mortality in young children. We also recommend conducting rotavirus disease burden and cost–effectiveness studies to explore the benefits of introduction of rotavirus vaccine.

## Introduction

Diarrhoeal disease is the second leading cause of death in children under age 5 worldwide and is estimated to kill 700 000 children annually. (*1*, *2*) In the past few decades, efforts to improve diarrhoea prevention and management have significantly reduced the number of diarrhoeal deaths in developed countries. (*1*) However, diarrhoeal disease remains a significant disease burden and one of the leading causes of death in children under age 5 in less developed countries where there are ongoing problems with poor nutrition and sanitation and access to safe water. (*1*, *3*)

Acute watery diarrhoea (AWD), which can last several hours to several days, is defined as the passage of three or more loose or liquid stools within 24 hours. (*4*) Severe and fatal diarrhoea occur when depleted body fluids are not replenished, leading to severe dehydration. The major causes of AWD in less developed countries include bacterial, viral and parasitic pathogens spread by the fecal–oral route through contaminated food, water or fomites as a result of poor hygiene. (*5*) In children under age 5, rotavirus is the leading cause of AWD globally and contributes to 38.3% of the hospitalization for diarrhoeal diseases. (*5*)

In the Lao People's Democratic Republic, it has been estimated that 11% of the under5 mortality is due to diarrhoea. (*6*) In 2004, AWD was added to the list of the National Notifiable Diseases, and the epidemiological trends of AWD are monitored through an indicator-based surveillance (IBS) system. In this system, all AWD cases presenting to health facilities are reported weekly to the National Center for Laboratory and Epidemiology (NCLE). In 2008, an electronic reporting system, Lao People's Democratic Republic Early Warning and Response Network (LAOEWARN), was introduced and replaced the previous paper-based reporting system, as described previously. (*7*) LAOEWARN is an Access-based (Microsoft Corporation, Redmond, WA, USA) electronic database in which weekly reports of all 17 nationally notifiable diseases and syndromes, including AWD, are entered and stored. This system also generates automated early warning alerts. To monitor the etiology of diarrhoeal diseases, eight diarrhoea sentinel surveillance sites were established in Vientiane Capital in 2013. These sites collect stool specimens from diarrhoea patients; specimens from patients under age 5 collected during the dry winter season (from October to April) are sent to NCLE for rotavirus testing.

Since the introduction of LAOEWARN, there has been no formal analysis of AWD data over time, and little is known about the geographic distribution of AWD in the Lao People's Democratic Republic. In this study, we aim to describe the trends of AWD in the Lao People's Democratic Republic from 2009 to 2013 with a particular focus on the prevalence of rotavirus-related morbidity in children under age 5.

## Methods

We conducted a review of AWD notification data by person, place and time. We also analysed the laboratory results from all eight diarrhoea sentinel sites in the Lao People's Democratic Republic.

### Data source

#### Case definition

An AWD case was defined as any patient passing loose or watery stools three or more times within 24 hours, which is consistent with World Health Organization (WHO) guidelines. (*4*)

#### IBS case-based data and LAOEWARN aggregated data for AWD

IBS passively collects aggregated and case-based reports of AWD cases from a total of 1115 health-care facilities nationwide. Weekly, information of AWD cases is first reported from 949 health centres and 142 hospitals to their district health offices by fax, telephone or in person and compiled into a line list. Together with the compiled data from 17 provincial and seven central hospitals, information is reported to the provincial level by e-mail or fax. All the line lists are then compiled into an overall AWD line list (case-based data) that is e-mailed to NCLE, and a record of the aggregated AWD cases that is entered into LAOEWARN (aggregated data). The case-based data are in Excel format (Microsoft Corporation, Redmond, WA, USA) and contain data of demographics, location, onset date and the date of hospitalization of the cases. The aggregated data contain four variables: number of cases and deaths, location and the week of reporting. Both case-based and aggregated data in 2009–2013 were analysed in this study.

#### Diarrhoea laboratory sentinel surveillance

We reviewed the 2013 laboratory data from all eight diarrhoea sentinel surveillance sites, which are all based in Vientiane Capital. Patients under age 5 with acute diarrhoea presenting to one of these sites during the dry season (October to April) were tested for rotavirus infections. Stool specimens collected from these cases were sent to NCLE for rotavirus rapid testing (Standard Diagnostics Inc., Gyeonggi-do, Republic of Korea). On rare occasions, some specimens were not tested due to the unavailability of technicians or test kits.

### Data analysis

We conducted descriptive analysis of AWD data using Excel. Overall, age- and sex-specific incidence rates were calculated for individual years using population figures from NCLE. Relative risks and 95% confidence intervals (CI) were calculated using Epi Info version 7.1.4 (Centers for Disease Control and Prevention, Atlanta, GA, USA). We mapped the geographical distribution of case notification rates by location using ArcView GIS version 3.2a (ESRI, Redlands, CA, USA).

Since all data collected were de-identified secondary data, ethical approval was waived.

## Results

### Descriptive epidemiology

**Fig. 1:**
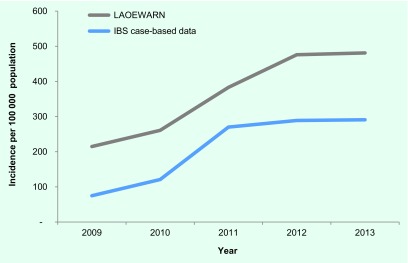

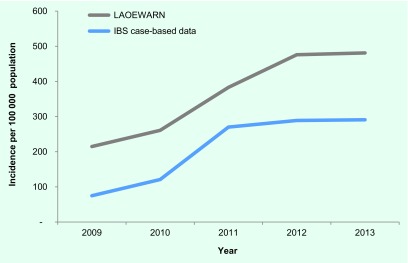
Incidence of AWD cases using LAOEWARN and IBS case-based data, Lao People's Democratic Republic, 2009–2013

AWD, acute watery diarrhoea; IBS, Indicator-based surveillance; LAOEWARN, Lao People's Democratic Republic Early Warning And Response Network.

Between 2009 and 2013, a total of 117 277 and 67 755 AWD cases were reported through LAOEWARN and IBS case-based reports, respectively. The incidence ranged for the LAOEWARN data (Fig. 1) between 215 and 476 per 100 000 population and increased from 2009 to 2012 when it levelled off. IBS case-based data follow the same trend but yield consistently lower AWD incidence than LAOEWARN aggregated data.

**Fig. 2:**
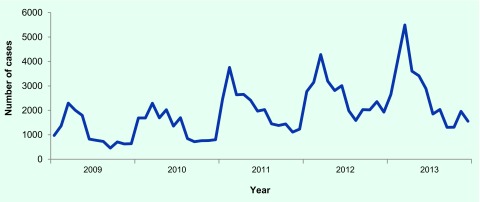

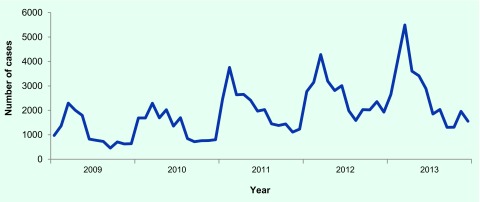
Secular and seasonal trends of AWD cases using LAOEWARN data, Lao People's Democratic Republic, 2009–2013*

AWD, acute watery diarrhoea; LAOEWARN, Lao People's Democratic Republic Early Warning And Response Network.

**Fig. 3:**
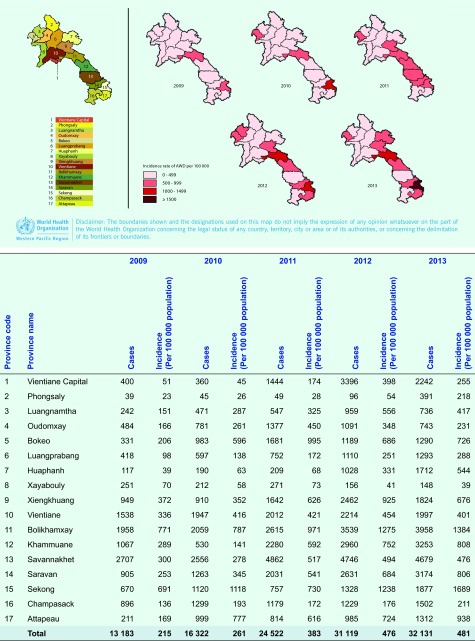
Geographical distribution of AWD incidence using LAOEWARN data, Lao People's Democratic Republic, 2009–2013 **Table:** 

Through LAOEWARN, a total of 37 deaths were also reported. Case notification had a seasonal trend and peaked around March in all years (except 2011 where cases peaked in February) (**Fig. 2**); a quarter (*n* = 30 149, 25.7%) of the total cases occurred during March–April. Reported incidence increased over the study period in almost all provinces (**Fig. 3**) with the highest incidence rates reported from Bolikhamxay  (771/100 000 to 1384/100 000 population) and Sekong provinces (691/10 000 to 1689/10 000 population).

Province Code	Province Name	2009		2010		2011		2012		2013	
		cases	Incidence (Per 100 000 population)	cases	Incidence (Per 100 000 population)	cases	Incidence (Per 100 000 population)	cases	Incidence (Per 100 000 population)	cases	Incidence (Per 100 000 population)
1	Vientiane capital	400	51	360	45	1444	174	3396	398	2242	255
2	Phongsaly	39	23	45	26	49	28	96	54	391	218
3	Luangnamtha	242	151	471	287	547	325	959	556	736	417
4	Oudomxay	484	166	781	261	1377	450	1091	348	743	231
5	Bokeo	331	206	983	596	1681	995	1189	686	1290	726
6	Luangprabang	418	98	597	138	752	172	1110	251	1293	288
7	Huaphanh	117	39	190	63	209	68	1028	331	1712	544
8	Xayabouly	251	70	212	58	271	73	156	41	148	39
9	Xiengkhuang	949	372	910	352	1642	626	2462	925	1824	676
10	Vientiane	1538	336	1947	416	2012	421	2214	454	1997	401
11	Bolikhamxay	1958	771	2059	787	2615	971	3539	1275	3958	1384
12	Khammuane	1067	289	530	141	2280	592	2960	752	3253	808
13	Savannakhet	2707	300	2556	278	4862	517	4746	494	4679	476
14	Saravan	905	253	1263	345	2031	541	2631	684	3174	806
15	Sekong	670	691	1120	1118	757	730	1328	1238	1877	1689
16	Champasack	896	136	1299	193	1179	172	1229	176	1502	211
17	Attapeau	211	169	999	777	814	616	985	724	1312	938
-	Total	13 183	215	16 322	261	24 522	383	31 119	476	32 131	481

AWD, acute watery diarrhoea.

**Fig. 4:**
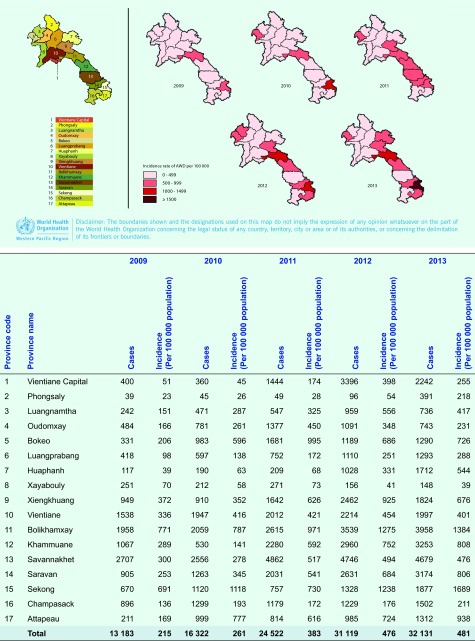

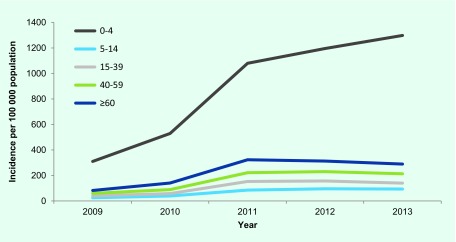
Age-specific incidence of AWD using IBS case-based data, Lao People's Democratic Republic, 2009–20

AWD, acute watery diarrhoea; IBS, Indicator-based surveillance.

Among the case-based data, 35 709 (52.7%) were from children under age 5. The incidence increased from 310/100 000 population in 2009 to 1298/100 000 population in 2013 (**Fig. 4**). For the cases under age 5, the majority (*n* = 26 722, 74.8%) were aged 0–24 months. The overall relative risk of the under 5 age group compared to others was 7.81 (95% CI: 7.69–7.93, *P* < 0.0001) (**Table 1**) and remained consistent year-to-year throughout the study period (range: 7.00–8.82). The median age of AWD cases for males was 2.1 years, and 7.0 years for females. Males under age 5 were more at risk (RR = 1.28, 1.25–1.31, *P* < 0.0001) for AWD than females under 5, but males aged 5 years or older were less at risk (RR = 0.87, 0.85–0.89, *P* < 0.0001) than females over 5 (**Table 2**). This pattern also remained consistent throughout the study period (under 5 years: RR range = 1.24–1.33; 5 years or older: RR range = 0.84–0.89).

**Table 1:** Incidence of AWD distribution by age using IBS case-based data, Lao People's Democratic Republic, 2009–2013

Age group	Incidence and RR	Year					Overall
		2009	2010	2011	2012	2013	
< 5 years old	Total population*	764 940	781 532	798 598	816 152	834 211	3 995 433
	Total AWD cases	2370	4141	8619	9750	10 829	35 709
	Incidence of AWD (per 100 000 population)	310	530	1079	1195	1298	894
< 5 years old (reference)	Total population*	5 363 262	5 478 325	5 596 620	5 718 253	5 843 329	27 999 789
	Total AWD cases	2216	3449	8631	9149	8601	32 046
	Incidence of AWD (per 100 000 population)	41	63	154	160	147	114
	RR	7.50	8.41	7.00	7.47	8.82	7.81
	95% CI	7.08–7.95	8.04–8.80	6.79–7.21	7.26–7.68	8.57–9.07	7.69–7.93
	*P*-value	< 0.0001	< 0.0001	< 0.0001	< 0.0001	< 0.0001	< 0.0001

AWD, acute water diarrhoea; IBS, indicator-based surveillance; CI, confidence intervals; RR, relative risk.

*Data source: Official population data, Ministry of Health, Lao People's Democratic Republic

**Table 2:** Incidence of AWD distribution by age and sex using IBS case-based data, Lao People's Democratic Republic, 2009–2013

Age group	Sex	Incidence and RR			Year			Overall
			2009	2010	2011	2012	2013	
< 5 years old	Male	Total population*	388 590	397 018	405 688	414 605	423 779	2 029 680
		Total AWD cases	1345	2323	4862	5648	6158	20 336
		Incidence of AWD (per 100 000 population)	346	585	1198	1362	1453	1002
	Female (reference)	Total population*	376 350	384 514	392 910	401 547	410 432	1 965 753
		Total AWD cases	1025	1818	3757	4101	4671	15 372
		Incidence of AWD (per 100 000 population)	272	473	956	1021	1138	782
	-	RR	1.27	1.24	1.25	1.33	1.28	1.28
		95% CI	1.17–1.38	1.16–1.32	1.20–1.31	1.28–1.39	1.23–1.33	1.25–1.31
		P-Value	< 0.0001	< 0.0001	< 0.0001	< 0.0001	< 0.0001	< 0.0001
≥ 5 years old	Male	Total population*	2 649 451	2 706 293	2 764 730	2 824 817	2 886 605	13 831 896
		Total AWD cases	1031	1596	3994	4117	3989	14 727
		Incidence of AWD (per 100 000 population)	39	59	144	146	138	106
	Female (reference)	Total population*	2 713 811	2 772 032	2 831 890	2 893 436	2 956 724	14 167 893
		Total AWD cases	1185	1853	4637	5032	4612	17 319
		Incidence of AWD (per 100 000 population)	44	67	164	174	156	122
	-	RR	0.89	0.88	0.88	0.84	0.89	0.87
		95% CI	0.82–0.97	0.83–0.94	0.85–0.92	0.80–0.87	0.85–0.92	0.85–0.89
		*P*-Value	< 0.0001	< 0.001	< 0.0001	< 0.0001	< 0.0001	< 0.0001

AWD, acute watery diarrhoea; IBS, indicator-based surveillance; CI, confidence intervals; RR, relative risk.

*Data source: Official population data, Ministry of Health, Lao People's Democratic Republic; sex ratios for < 5 years old (male = 50.8%, female = 49.2%) and ≥ 5 years old (male = 49.4%, female = 50.6%) used for the calculation were derived from 2005 Census.

### Laboratory surveillance

**Fig. 5:**
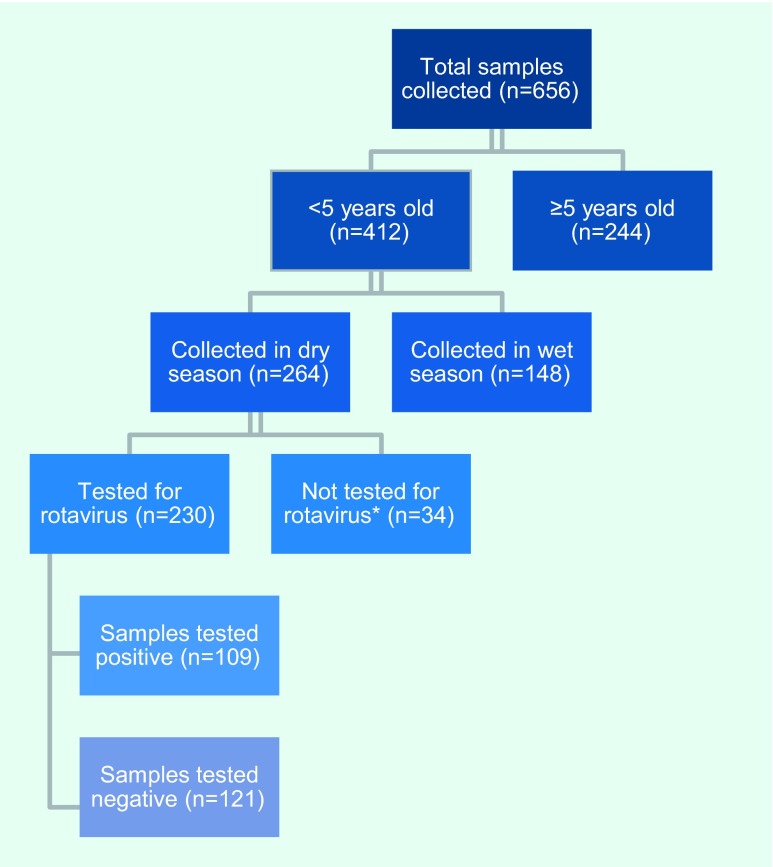

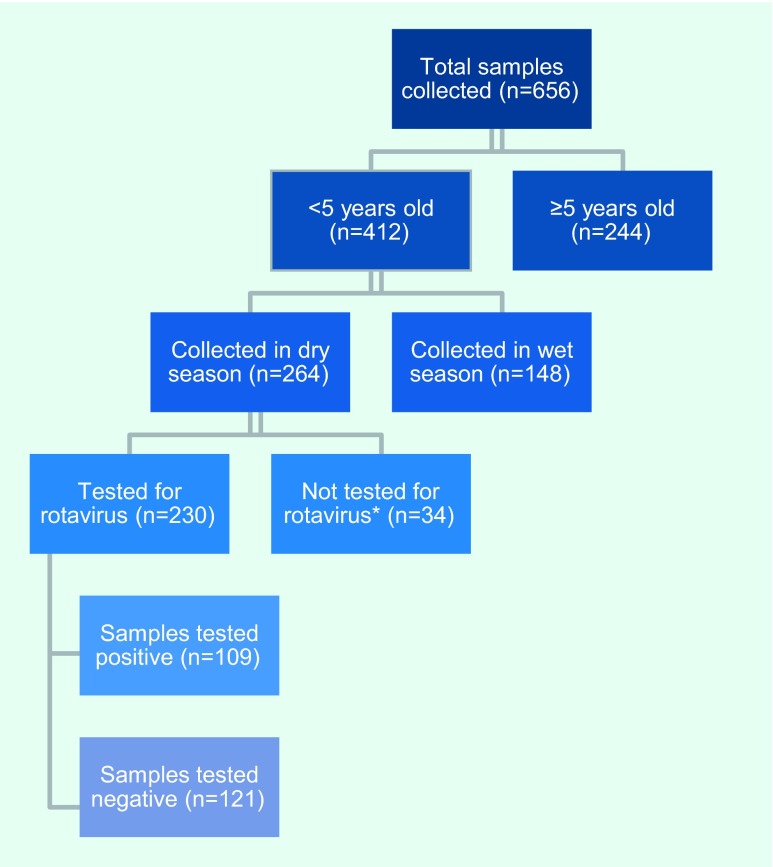
Flowchart of samples tested for rotavirus in all eight diarrheal sentinel sites, Lao People's Democratic Republic, 2013

In 2013, a total of 656 stool specimens were submitted to the laboratory sentinel surveillance system (**Fig. 5**). The number of specimens peaked in March and April in 2013 (180/656, 27.4%). The majority (412/656, 62.8%) were from children under 5 years; half (331/656, 50.5%) of the cases were aged 0–24 months. Among the 412 stool specimens from children under 5, 264 (64.1%) were collected during the dry season. NCLE tested 230 (87.1%) of them for rotavirus, and 109 (47.4%) tested positive.

*On rare occasions, some specimens were not tested due to the unavailability of technician or test kit stock.

## Discussion

In this study, we investigated the trends of AWD from the national IBS system from 2009 to 2013. Although a health facility-based IBS system can only capture cases that seek health care, and the captured data depend on many factors such as each case’s condition, financial situation and distance from health facilities, it is useful for monitoring disease trends over time.

The reported number of AWD cases from the case-based data was lower than that in the LAOEWARN data, presumably due to underreporting given the higher workload associated with additional information required as well as needing a stable Internet to send spreadsheets. Also, the number of fatal AWD cases is much lower than the published estimates for the Lao People's Democratic Republic. (*6*) It is likely that a high number of AWD deaths occurred outside of health-care facilities. Possible reasons may include that those living in rural areas have limited health-care access and the cultural practice of transporting critically ill patients back home before death.

We showed that AWD notification is the highest early in life as documented previously. (*8*) We identified the under5 population at the greatest risk for AWD, with annual relative risk consistently ranging from seven to almost nine times higher than those 5 years or older. With nearly half of AWD in the under5 group attributed to rotavirus, this pattern could be explained by the protective immunity developed after initial exposure to rotavirus early in life. (*9*) It is well known that breastfeeding can significantly reduce morbidity and mortality due to AWD. (*2*) WHO recommends that mothers breastfeed exclusively up to six months and continue breastfeeding up to two years. (*10*) However, in the Lao People's Democratic Republic, only 40.4% of the mothers breastfeed exclusively up to six months; 40.0% of the mothers breastfeed up to two years. (*11*) Promoting breastfeeding practice could be one way to reduce the AWD incidence in these young children.

We identified males under age 5 being more at risk, a trend that also is seen in other studies including those conducted in Indonesia and Guinea-Bissau; however, the reason is unclear. (*12*, *13*) A similar sex trend was seen among under5 diarrhoea cases in a large nationwide household survey conducted in 2011–2012 in the Lao People's Democratic Republic regardless of whether they sought health care; (*11*) therefore, a difference in health-care-seeking behaviour for the two sexes is unlikely to offer the full explanation.

Sekong and Bolikhamxay provinces have the highest incidence of AWD. Although the reason for this is unclear, this finding is consistent with the national event-based surveillance data where AWD outbreaks most frequently occur in these two provinces (unpublished, NCLE, 2016). Based on a national social indicator survey conducted in 2011–2012, (*11*) Sekong province has one of the highest prevalences of open defecation (52.1% of the households), and Bolikhamxay is known to have inadequate water treatment of unimproved water sources. These could be potential contributing factors explaining the higher disease incidences. (*11*)

Our findings indicated that rotavirus is the etiology for almost half of the under5 AWD cases during the dry winter season, consistent with worldwide estimates of 39.4% of diarrhoeal episodes in this age group being attributed to rotavirus. (*5*) Based on existing literature describing the pattern of rotavirus transmission in other tropical countries, we believe the peak AWD notifications in the dry winter seasons may be primarily driven by rotavirus. The seasonality of rotavirus has been well studied and is known to vary by region and climate. (*14*) It has been shown in similar tropical countries that disease transmission for rotavirus increases with decreasing humidity and temperature; (*15*, *16*) previous studies have hypothesized that the dried fecal matter containing rotavirus may become airborne during this time, driving disease transmission. (*16*, *17*)

There are several limitations in this study. First, there may be a degree of underascertainment from cases who visited private health facilities and were not reported to this system. Also, this system cannot capture cases who sought traditional healers or self-medication instead of formal health care or deaths which occurred outside the health-care facilities, leading to underestimates of the incidence and mortality rate. Second, secular trends of increasing notification of AWD in the Lao People's Democratic Republic may represent the increasing sensitivity of AWD surveillance associated with the electronic LAOEWARN system rather than an underlying changes in the trend of AWD. The representativeness of etiological data in this study may be limited because all sentinel sites were located in Vientiane Capital; specimens were only tested for rotavirus during the dry season and we only examined rotavirus rapid test data in 2013.

To our knowledge, this is the first time the epidemiological trend of AWD has been studied in the Lao People's Democratic Republic. Based on the results, we encourage new mothers to breastfeed for up to two years per the WHO recommendation to reduce incidence of AWD in young children. (*2*, *10*) We also recommend integrating hygiene and sanitation health education into nursery and primary schools, so children can bring their knowledge home to benefit the entire family. Many questions remain that are critical to the planning of targeted control and prevention strategies for AWD. Therefore we recommend: 1) conducting further study of diarrhoea-associated mortality, such as through community-based verbal autopsy studies to capture deceased cases outside the health facilities; 2) exploring risk factors for AWD during dry seasons in different regions; 3) expanding laboratory sentinel sites to increase geographic diversity; and 4) conducting rotavirus disease burden and cost–effectiveness studies to explore introducing rotavirus vaccine into the routine immunization schedule.
